# The influence of athletic identity, coach-athlete relationship quality, and peer climate on emotional resilience in injury-prone sports

**DOI:** 10.3389/fpsyg.2026.1791069

**Published:** 2026-07-15

**Authors:** Songmao Yu, Haozhun Luo

**Affiliations:** 1 School of Physical Education, Southwest University, Chongqing, China; 2 College of Economics and Management, Southwest University, Chongqing, China

**Keywords:** athletic identity, coach–athlete relationship quality, emotional resilience, injury-prone sports, peer climate, psychological adaptation

## Abstract

**Introduction:**

Athletes competing in injury-prone sports are routinely exposed to physical risk and psychological strain, making psychological adaptation a critical yet underexplored issue in sport psychology. Drawing on identity-based perspectives, conservation of resources theory, and self-determination theory, this study examined whether athletic identity influences psychological adaptation through emotional resilience and whether coach–athlete relationship quality and peer climate strengthen this process.

**Methods:**

A cross-sectional survey was conducted among 482 athletes participating in injury-prone sports in China. Data were collected using validated self-report questionnaires and analyzed using partial least squares structural equation modeling (PLS-SEM). Mediation and moderated mediation analyses were performed to examine the relationships among athletic identity, emotional resilience, psychological adaptation, coach–athlete relationship quality, and peer climate.

**Results:**

Athletic identity was positively associated with emotional resilience, which in turn significantly predicted psychological adaptation, supporting a partial mediation effect. Coach–athlete relationship quality and peer climate both positively moderated the relationship between athletic identity and emotional resilience. Conditional indirect effect analyses further demonstrated that the mediating role of emotional resilience was significantly stronger under conditions of high-quality coach–athlete relationships and supportive peer climates.

**Discussion:**

The findings suggest that athletic identity contributes to psychological adaptation by enhancing emotional resilience, and that this process is strengthened by supportive interpersonal environments. These results underscore the importance of considering both individual identity resources and social-contextual factors when promoting athletes’ psychological adaptation in injury-prone sports.

## 1 Introduction

Psychological adaptation has become a central concern in contemporary sport psychology (Quan et al., 2025), particularly in competitive contexts characterized by persistent physical risk and uncertainty. Athletes engaged in injury-prone sports are not only required to manage physical recovery but must also navigate emotional disruption, identity threats, and ongoing performance pressure. While previous research has extensively examined injury incidence and rehabilitation outcomes (Krutsch et al., 2025; Rojas-Valverde et al., 2025), considerably less attention has been paid to the psychological processes that enable athletes to maintain adaptive functioning in the face of recurrent injury risk. This gap is notable, given growing evidence that psychological adaptation plays a critical role in long-term wellbeing, sustained engagement, and performance continuity in demanding sport environments (Rubio et al., 2021; Samuel et al., 2024).

Building on this concern, scholars have increasingly emphasized the role of self-concept and emotional regulation in shaping athletes’ responses to stress and adversity. Identity-based perspectives suggest that how individuals define themselves can profoundly influence their emotional experiences and coping behaviors under threat (Campo et al., 2019). In sport settings, identification with the athlete role has been linked to motivation, persistence, and goal commitment, but it has also been associated with vulnerability when performance or participation is disrupted (Shaikh and Forneris, 2024). This duality has generated mixed empirical findings, leaving unresolved questions regarding when and how identity-related processes facilitate, rather than hinder, psychological adaptation in high-risk sport contexts (Kabak and Baş, 2026).

At the same time, resilience has emerged as a key construct for understanding adaptive psychological functioning under stress. Contemporary resilience research conceptualizes resilience not merely as resistance to adversity, but as a dynamic capacity to recover emotionally and maintain psychological balance in the face of ongoing challenges (Köroglu et al., 2025; Marheni et al., 2024). Within sport psychology, resilience has been linked to wellbeing, sustained performance, and positive adjustment following setbacks (Husain et al., 2024). However, resilience has rarely been examined as a mechanism through which identity-related processes influence broader psychological adaptation, particularly in environments where injury risk is chronic rather than episodic (Mastroianni et al., 2025).

Beyond individual-level processes, increasing attention has been directed toward the social contexts in which athletes are embedded. Relational environments, including interactions with coaches and peers, have been shown to shape athletes’ motivation, emotional experiences, and psychological health (Santos et al., 2025). From a self-determination theory perspective, supportive social relationships satisfy fundamental psychological needs, thereby enabling individuals to regulate emotions more effectively and respond adaptively to stress (Ryan and Deci, 2000). Despite this growing body of work, limited research has examined how relational factors condition the psychological pathways through which self-concept translates into adaptive outcomes.

Against this backdrop, the present study addresses several important gaps in the literature. First, it advances identity-based research by shifting attention from direct associations toward the psychological mechanisms that explain how identity contributes to adaptation in injury-prone sport contexts. Second, it integrates emotional resilience as a central explanatory process linking self-concept to adaptive psychological functioning, thereby extending resilience research into identity-driven frameworks. Third, it adopts a contextualized perspective by examining how coach–athlete relationship quality and peer climate jointly shape the strength of this mechanism. By proposing and testing a moderated mediation framework, the study offers a more nuanced and ecologically valid understanding of psychological adaptation in sport.

In doing so, the study makes a threefold theoretical contribution. It reconceptualizes athletic identity as a conditional psychological resource rather than a uniformly adaptive or maladaptive construct, clarifies the role of emotional resilience as a key mechanism of psychological adaptation, and highlights the critical importance of relational environments as boundary conditions for identity-based emotional processes. Collectively, these contributions extend existing theory and provide a more integrated account of how athletes adapt psychologically in injury-prone sport settings.

Previous research has increasingly emphasized that athletes’ adaptive psychological functioning is shaped not only by individual identity-related resources, but also by emotional and relational processes embedded within the broader sport context (Campo et al., 2019; Gupta and McCarthy, 2022). Athletic identity has been associated with persistence, emotional regulation, coping effectiveness, and psychological continuity during periods of stress, injury, and rehabilitation (Christino et al., 2021; Liu and Noh, 2024; McGinley et al., 2022). Likewise, emotional resilience has been consistently linked to wellbeing, recovery from setbacks, sustained motivation, and adaptive functioning in demanding sport settings (Husain et al., 2024; Junnarkar et al., 2021; Mastroianni et al., 2025). Beyond these individual-level resources, relational environments characterized by supportive coach–athlete interactions and positive peer climates have also been shown to strengthen athletes’ emotional functioning, motivation, and psychological stability under pressure (Karayel et al., 2024; Li et al., 2025; Zhang et al., 2023). Collectively, this body of literature suggests that psychological adaptation in injury-prone sports is likely to emerge through the interaction of personal identity resources, emotional resilience capacities, and supportive social-contextual conditions.

Drawing on identity-based perspectives, conservation of resources theory (Hobfoll, 1989), and self-determination theory (Ryan and Deci, 2000), the proposed framework integrates individual psychological resources with relational contextual factors to provide a more comprehensive explanation of adaptive psychological functioning in high-risk sport environments.

Therefore, the present study seeks to advance understanding of psychological adaptation in injury-prone sports through the following objectives:

Primary objective: To examine the influence of athletic identity on psychological adaptation among athletes participating in injury-prone sports.
Secondary objective 1: To investigate whether emotional resilience mediates the relationship between athletic identity and psychological adaptation.
Secondary objective 2: To examine whether coach–athlete relationship quality strengthens the relationship between athletic identity and emotional resilience.
Secondary objective 3: To examine whether peer climate strengthens the relationship between athletic identity and emotional resilience.
Secondary objective 4: To determine whether the indirect effect of athletic identity on psychological adaptation through emotional resilience varies across different levels of coach–athlete relationship quality and peer climate.

Based on these objectives, seven hypotheses (H1–H7b) are developed and tested in the subsequent sections.

## 2 Hypotheses

### 2.1 Athletic identity and emotional resilience

The present study expects that athletic identity plays a meaningful role in shaping athletes’ emotional responses to stress in injury-prone sport contexts. Identity-based research suggests that a strong self-definition provides individuals with purpose, structure, and motivational clarity, which can facilitate emotional stability under adverse conditions (Jiao et al., 2024). Empirical evidence indicates that athletes with a salient athletic identity demonstrate greater persistence and psychological engagement when facing performance challenges (Shinnick, 2024). Other studies have shown that identity coherence supports adaptive coping and emotional regulation in high-demand environments (Christino et al., 2021; Scheadler et al., 2021). Research in rehabilitation contexts further suggests that athletes who remain psychologically connected to their athletic role report more positive emotional adjustment during recovery periods (McGinley et al., 2022). Similarly, identity continuity has been associated with enhanced emotional recovery following setbacks in competitive sport (Liu and Noh, 2024). From a conservation of resources perspective, athletic identity may serve as a valuable psychological resource that enhances athletes’ capacity to regulate emotions and recover from adversity in high-risk sport environments (Hobfoll, 1989). Taken together, these findings suggest that athletic identity can foster emotional resilience rather than emotional vulnerability in injury-prone sports. Therefore, the study proposes:

H1: Athletic identity is positively related to emotional resilience.

### 2.2 Emotional resilience and psychological adaptation

The study further proposes that emotional resilience is a critical determinant of psychological adaptation in injury-prone sport environments. Emotional resilience reflects an individual’s capacity to recover from stress and maintain psychological balance when confronted with adversity (Scheadler et al., 2021). Prior research has demonstrated that resilient athletes exhibit better emotional control, reduced psychological distress, and more adaptive cognitive appraisals under pressure (Köroglu et al., 2025; Marheni et al., 2024). Studies have also linked resilience to wellbeing, sustained motivation, and functional adjustment in high-performance sport contexts (Junnarkar et al., 2021; Oguntuase and Sun, 2022). Evidence from injury research indicates that emotionally resilient athletes are more likely to maintain psychological engagement and positive outlooks during rehabilitation (Ernst et al., 2022; Mastroianni et al., 2025). Moreover, resilience has been shown to predict adaptive responses to uncertainty and performance disruption (Gupta and McCarthy, 2022). These findings collectively suggest that emotional resilience enables athletes to translate stress exposure into adaptive psychological outcomes rather than maladaptive responses. Accordingly, the study hypothesizes:

H2: Emotional resilience is positively related to psychological adaptation.

### 2.3 Mediating role of emotional resilience

Beyond indirect pathways, the study anticipates a direct association between athletic identity and psychological adaptation. Research has shown that a strong athletic identity is associated with higher levels of engagement, goal commitment, and perceived meaning in sport participation (Powell et al., 2024; Roberts et al., 2023). Athletes who strongly identify with their sport role tend to interpret challenges as integral to their identity rather than as threats to self-worth (Edison et al., 2021). Empirical studies have demonstrated that identity salience predicts psychological persistence and adjustment during periods of disruption (Hayes et al., 2023; Hu et al., 2021; Liu and Noh, 2024; McGinley et al., 2022). Other work suggests that identity clarity supports adaptive self-regulation and emotional stability in competitive contexts (Albouza et al., 2022; García et al., 2023). At the same time, scholars have cautioned that excessively rigid athletic identities may become psychologically vulnerable when athletes experience severe injury, prolonged absence from competition, or threats to performance continuity (Brewer and Chatterton, 2024; Lochbaum et al., 2022). Under such conditions, athletes may experience identity disruption, emotional distress, and difficulties adjusting to temporary or permanent changes in sport participation. Nevertheless, contemporary research increasingly suggests that athletic identity is more likely to facilitate psychological adaptation when supported by effective coping resources and emotionally supportive environments (McGinley et al., 2022; Schmid et al., 2024). Although excessive identity foreclosure may pose risks, contemporary research increasingly recognizes that identity strength can also promote psychological adaptation when supported by appropriate coping resources (Christino et al., 2021; Steger and Harris, 2022). Based on this reasoning, the study proposes:

H3: Athletic identity is positively related to psychological adaptation.

Building on the above relationships, the study proposes that emotional resilience serves as a psychological mechanism linking athletic identity to psychological adaptation. Identity-based models suggest that self-concept influences outcomes by shaping emotional and cognitive responses to stress (Shinnick, 2024). Consistent with conservation of resources theory, identity-related psychological resources may promote adaptive functioning indirectly by strengthening emotional resilience under stressful conditions (Hobfoll, 1989). Empirical research indicates that identity-related resources facilitate emotional regulation and recovery following setbacks (Liu and Noh, 2024; Renton et al., 2021). Studies in sport resilience research have shown that emotional resilience mediates the relationship between stress exposure and adaptive outcomes (Levillain et al., 2024; Secer et al., 2025). Additionally, resilience has been identified as a key explanatory process connecting personal strengths to psychological wellbeing (Junnarkar et al., 2021). Collectively, this evidence suggests that athletic identity contributes to psychological adaptation by strengthening athletes’ emotional resilience. Thus, the study hypothesizes:

H4: Emotional resilience mediates the relationship between athletic identity and psychological adaptation.

### 2.4 Moderating role of coach–athlete relationship quality

The present study proposes that the psychological meaning athletes derive from their athletic identity may not be uniform across relational contexts. Within sport settings, the coach–athlete relationship represents a primary interpersonal environment through which emotional experiences are shaped. High-quality coach–athlete relationships have been conceptualized as involving mutual trust, commitment, and coordinated interaction, all of which carry psychological significance for athletes’ emotional functioning (Karayel et al., 2024). When such relational conditions are present, athletes are more likely to experience emotional security and psychological support, which can facilitate adaptive emotional responses under stress (Zhang et al., 2023). Empirical work has shown that athletes embedded in supportive coaching relationships report fewer maladaptive emotional outcomes and greater psychological stability in demanding sport contexts (Llanos-Muñoz et al., 2023; McManama O’Brien et al., 2021). Conversely, strained coach–athlete relationships have been linked to emotional exhaustion and psychological strain, particularly under performance pressure (Karayel et al., 2024). Furthermore, Aronen et al. (2021) suggest that coaches’ interpersonal behaviors influence how athletes regulate emotions and respond to setbacks. Emotional functioning has further been shown to be sensitive to interpersonal stressors within the coaching environment, especially in high-demand sport settings (Zhichao et al., 2022). Self-determination theory further suggests that supportive coach–athlete relationships satisfy athletes’ psychological needs for relatedness and competence, thereby strengthening the positive emotional effects of athletic identity under stressful sport conditions (Ryan and Deci, 2000). Taken together, these findings suggest that coach–athlete relationship quality may subtly condition how athletic identity relates to emotional resilience rather than acting as a dominant explanatory factor. Accordingly, the study hypothesizes:

H5: Coach–athlete relationship quality moderates the relationship between athletic identity and emotional resilience.

### 2.5 Moderating role of peer climate

The study also considers whether the broader peer environment shapes the association between athletic identity and emotional resilience. In team and training settings, peers constitute a salient social reference group that influences emotional norms, evaluative standards, and coping behaviors. Prior research has demonstrated that peer-created motivational climates are psychologically meaningful and distinct from coach-driven environments (Li et al., 2025; Wei et al., 2025). Athletes operating within supportive peer climates tend to report more positive emotional experiences and greater psychological comfort during training and competition (Marheni et al., 2024). Qualitative evidence further illustrates that peer interactions play a central role in shaping how athletes experience pressure, failure, and recovery from setbacks (Hussain et al., 2023). These dynamics appear to extend across age groups and competitive levels, underscoring the persistent influence of peers in sport contexts (Li et al., 2025). Psychological adjustment has also been linked to the quality of the broader sport environment, including peer relationships and social norms (Haddow et al., 2021). Social-contextual research suggests that emotionally supportive peer environments can buffer stress responses and promote emotional stability (Cheng and Jiao, 2024). From a social identity perspective, group belonging and positive group climates may enhance individuals’ capacity to cope with threat and uncertainty (Campo et al., 2019). Self-determination theory further suggests that supportive coach–athlete relationships satisfy athletes’ psychological needs for relatedness and competence, thereby strengthening the positive emotional effects of athletic identity under stressful sport conditions (Ryan and Deci, 2000). From a social identity and self-determination perspective, supportive peer climates may reinforce athletes’ sense of belonging and emotional security, thereby enabling athletic identity to function more effectively as a psychological resource (Campo et al., 2019; Ryan and Deci, 2000). Collectively, this literature suggests that peer climate may shape the conditions under which athletic identity functions as an emotional resource. Therefore, the study hypothesizes:

H6: Peer climate moderates the relationship between athletic identity and emotional resilience.

## 3 Moderated mediation of emotional resilience

**FIGURE 1:**
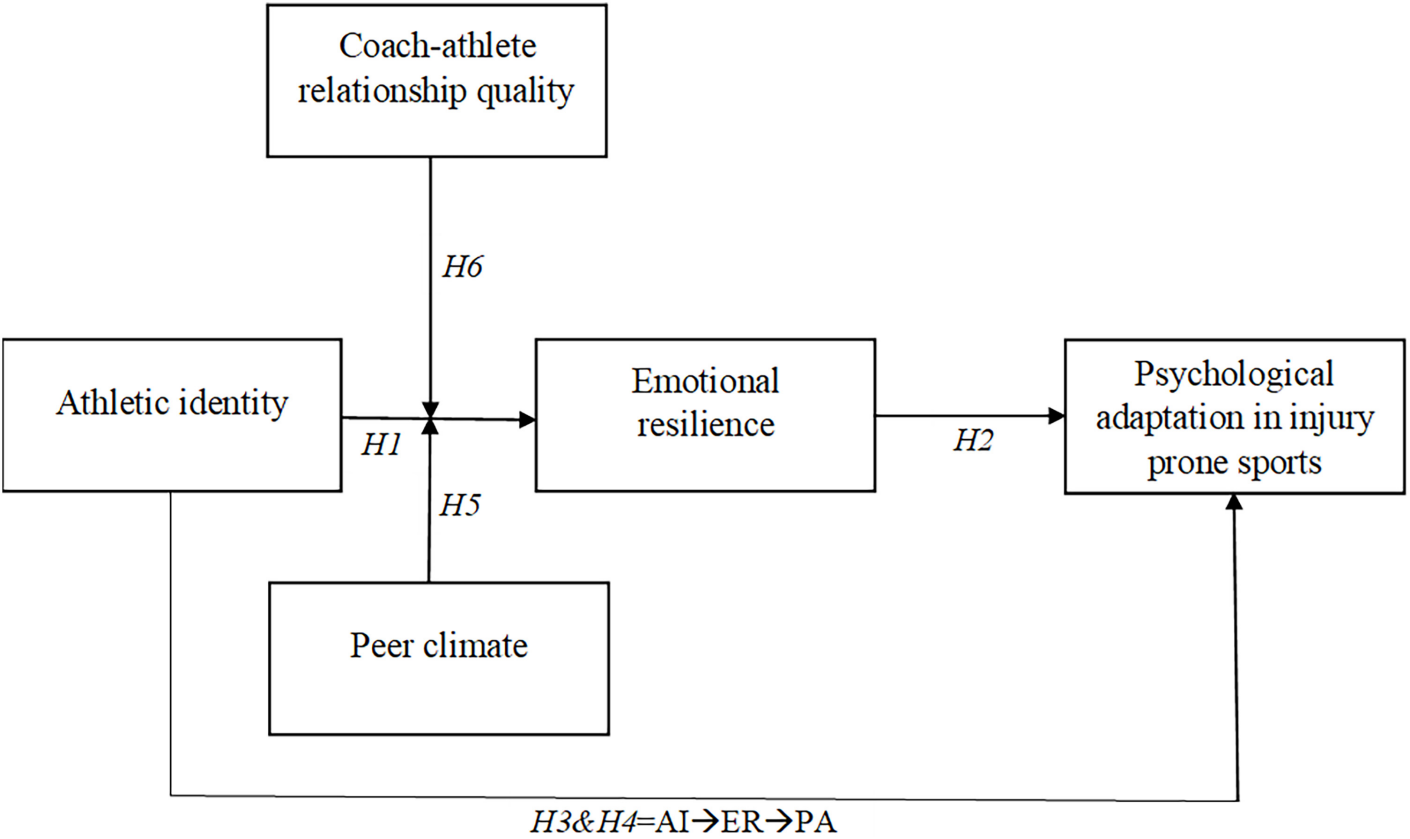
Conceptual model.

Integrating the proposed mediation and moderation processes, the study advances a conditional process model in which the indirect relationship between athletic identity and psychological adaptation via emotional resilience depends on relational context. Contemporary sport psychology research increasingly recognizes that resilience-related processes emerge from the interaction between individual characteristics and environmental conditions rather than from isolated personal traits (Ernst et al., 2022; Wei et al., 2025). Emotional resilience, in particular, has been shown to be shaped by social support and contextual resources in high-pressure sport environments (Gupta and McCarthy, 2022). Organizational and relational conditions have been linked to how athletes experience stress and translate psychological resources into adaptive outcomes (Powell et al., 2024). Evidence from social support research further indicates that relational contexts influence the effectiveness of personal coping mechanisms and adjustment processes (Cheng and Jiao, 2024; Haddow et al., 2021; Li et al., 2025). The logic of conditional indirect effects has been well-articulated in methodological frameworks that integrate mediation and moderation within a single explanatory model (Preacher et al., 2007). Similar integrative perspectives emphasize that indirect relationships may vary systematically across contextual conditions (Edwards and Lambert, 2007). From a resource-based viewpoint, personal resources are more likely to generate adaptive outcomes when supported by complementary contextual resources (Hobfoll, 1989). Environments that satisfy basic psychological needs may therefore strengthen the translation of emotional resilience into psychological adaptation (Ryan and Deci, 2000). In light of these perspectives, the study proposes that the mediating role of emotional resilience is contingent upon coach–athlete relationship quality and peer climate (Figure 1). Thus, the following hypothesis is advanced:

H7a&b: The indirect relationship between athletic identity and psychological adaptation via emotional resilience is moderated by coach–athlete relationship quality and peer climate.

## 4 Materials and methods

The study was conducted in China and focused on athletes participating in injury-prone sports, including contact, collision, and high-load individual and team sports that are commonly associated with elevated injury exposure and physical risk in sport psychology and sports medicine research (Brewer and Chatterton, 2024; Ernst et al., 2022; Mastroianni et al., 2025). A purposive sampling strategy was employed to ensure that participants had direct experience with the physical and psychological demands associated with heightened injury risk. This approach was considered appropriate because the research objectives required access to a specialized athletic population for whom issues of athletic identity, emotional resilience, and psychological adaptation are particularly salient. Participants were not required to have experienced a previous sport-related injury to be included in the study. Instead, eligibility was based on active participation in sports characterized by elevated physical risk and injury exposure, as the study focused on psychological adaptation within injury-prone sport environments rather than exclusively on post-injury rehabilitation experiences. Athletes without prior injuries were retained because they are similarly exposed to ongoing injury risk, performance pressure, and psychologically demanding sport conditions that may influence emotional resilience and adaptive psychological functioning. Athletes were recruited through universities, provincial sport teams, and professional training centers across several regions of China, with the assistance of coaches and sport administrators who facilitated access to eligible participants.

Data were collected using a structured self-administered questionnaire. The survey instrument was initially developed in English and subsequently translated into Mandarin Chinese using a back-translation procedure to ensure linguistic and conceptual equivalence. The translation and back-translation procedures were conducted by bilingual researchers proficient in both English and Mandarin Chinese. The translated version was subsequently reviewed by independent academic experts to verify linguistic accuracy, conceptual consistency, and contextual appropriateness for the Chinese sport setting. No items were removed during the adaptation process, although minor wording refinements were made to improve clarity and contextual relevance. Prior to participation, all respondents were informed of the academic purpose of the study, assured that their responses would remain anonymous and confidential, and advised that participation was voluntary. Questionnaires were distributed both in person during scheduled training sessions and electronically through secure online survey links circulated via official team communication platforms. This combined approach helped maximize participation while maintaining control over respondent eligibility and data quality.

A total of 620 questionnaires were distributed during the data collection period. After screening for incomplete responses, excessive missing data, and inconsistent answering patterns, 482 valid responses were retained for final analysis, resulting in an effective response rate of approximately 77.7%. Data collection was carried out over a 3-month period, from March to May 2024, which allowed sufficient time to reach athletes across different competitive levels and sport disciplines. The final sample size was considered adequate for the application of partial least squares structural equation modeling, particularly given the complexity of the proposed moderated mediation model.

The final sample comprised 482 athletes, of whom 58.1% were male and 41.9% were female. In terms of age, 24.7% of participants were younger than 20 years, 46.3% were between 20 and 25 years, 21.6% were between 26 and 30 years, and 7.4% were older than 30 years. Regarding competitive level, 39.8% competed at the university or collegiate level, 34.2% were affiliated with provincial or regional teams, and 26.0% participated at the national or semi-professional level. With respect to sport type, 42.5% of respondents were engaged in team-based injury-prone sports, while 57.5% participated in individual injury-prone sports. Additionally, 61.2% of the athletes reported experiencing at least one sport-related injury in the previous two years, indicating that the sample was well-suited for examining psychological adaptation processes in injury-prone sport contexts.

## 5 Measures

All study variables were measured using previously validated instruments that have been widely employed in sport psychology and related research contexts. To ensure contextual relevance, minor wording adaptations were made where necessary to reflect participation in injury-prone sports, following established scale adaptation practices.

### 5.1 Athletic identity

Athletic identity was measured using the Athletic Identity Measurement Scale originally developed by Brewer et al. (1993). This scale captures the extent to which individuals define themselves in terms of the athlete role and view sport participation as central to their self-concept. The instrument consists of seven items rated on a Likert-type scale. A representative item is “I consider myself an athlete.”

### 5.2 Emotional resilience

Emotional resilience was assessed using the Brief Resilience Scale developed by Smith et al. (2008). This scale focuses specifically on individuals’ perceived ability to recover from stress and emotional setbacks, making it particularly suitable for athletes exposed to injury risk and performance-related adversity. The scale comprises six items, with sample items including “I tend to bounce back quickly after hard times.”

### 5.3 Coach-athlete relationship quality

Coach–athlete relationship quality was measured using the Coach–Athlete Relationship Questionnaire developed by Jowett and Ntoumanis (2004). Grounded in the 3Cs conceptualization of the coach–athlete relationship, the instrument assesses perceived closeness, commitment, and complementarity within the dyadic relationship. The scale consists of eleven items, with a sample item being “I feel close to my coach.”

### 5.4 Peer climate

Peer climate was assessed using the Peer Motivational Climate in Youth Sport Questionnaire developed by Ntoumanis and Vazou (2005). This instrument captures athletes’ perceptions of peer-created motivational cues and social norms within the team environment, including both supportive and maladaptive peer behaviors. The scale consists of twenty-one items, with a representative item being “On this team, teammates encourage each other to improve.”

Although the Peer Motivational Climate in Youth Sport Questionnaire was originally developed for youth sport settings, subsequent sport psychology research has applied similar peer climate dimensions across broader competitive and developmental contexts due to the continuing relevance of peer interactions in organized sport environments (Li et al., 2025; Wei et al., 2025). In the present study, the instrument was considered appropriate because peer influence, emotional support, and team-based motivational dynamics remain salient across both collegiate and competitive adult sport settings. Furthermore, the scale demonstrated satisfactory psychometric performance in the current sample, including strong internal consistency reliability and construct validity within the Chinese athletic context.

### 5.5 Psychological adaptation

Psychological adaptation in injury-prone sports was operationalized using the Mental Health Continuum–Short Form developed by Keyes (2006). This scale assesses emotional, psychological, and social wellbeing, collectively reflecting adaptive psychological functioning under conditions of stress and adversity. In the context of injury-prone sports, psychological adaptation is conceptualized as athletes’ capacity to maintain positive emotional, psychological, and social functioning despite exposure to physical risk, uncertainty, and performance-related stressors. Consistent with prior sport psychology and adaptation research (Rubio et al., 2021; Samuel et al., 2024), the Mental Health Continuum–Short Form was considered appropriate because its multidimensional assessment of emotional, psychological, and social wellbeing captures key indicators of adaptive psychological functioning under stressful conditions. Accordingly, the present study operationalizes psychological adaptation through athletes’ overall positive mental functioning and wellbeing within demanding sport environments. The instrument consists of fourteen items, with sample items including “During the past month, How often did you feel that your life has a sense of direction or meaning?”

## 6 Analytical strategy

The proposed research model was examined using partial least squares structural equation modeling (PLS-SEM), which is particularly suitable for theory development and prediction-oriented research involving complex models with mediation and moderation effects (Hair et al., 2019, 2021). PLS-SEM is appropriate in this study given the simultaneous examination of multiple latent constructs, indirect effects, and conditional (moderated) relationships, as well as its robustness to non-normal data distributions and suitability for behavioral research in applied settings such as sport psychology.

## 7 Results

The analysis followed the recommended two-step procedure, beginning with an assessment of the measurement model to establish indicator reliability, internal consistency reliability, convergent validity, and discriminant validity, followed by an evaluation of the structural model to test the hypothesized relationships (Hair et al., 2021). Bootstrapping with 5,000 resamples was employed to obtain bias-corrected confidence intervals and assess the significance of the direct, indirect, and interaction effects, consistent with recommended practices for mediation and moderated mediation analysis in PLS-SEM research (Hair et al., 2019). The conditional indirect effects were examined to determine whether the mediating role of emotional resilience varied as a function of coach–athlete relationship quality and peer climate, thereby allowing for a rigorous test of the proposed moderated mediation framework.

The measurement model was first assessed to establish internal consistency reliability, convergent validity, and discriminant validity. As shown in Table 1, Cronbach’s alpha values ranged from 0.861 to 0.904, exceeding the recommended threshold of 0.70, indicating satisfactory internal consistency reliability. Composite reliability values ranged from 0.903 to 0.929, further confirming the reliability of all latent constructs (Hair et al., 2021). Convergent validity was supported, as the average variance extracted (AVE) values for all constructs exceeded the minimum criterion of 0.50, ranging from 0.651 to 0.725, indicating that each construct explained more than half of the variance in its indicators.

**TABLE 1:** Measurement model evaluation.

Construct	CA	CR	AVE	AI	ER	CARQ	PC	PA
Athletic identity (AI)	0.883	0.915	0.685	0.828	–	–	–	–
Emotional resilience (ER)	0.861	0.903	0.651	0.463	0.807	–	–	–
Coach–athlete relationship quality (CARQ)	0.904	0.929	0.725	0.389	0.512	0.851	–	–
Peer climate (PC)	0.892	0.921	0.701	0.417	0.486	0.558	0.837	–
Psychological adaptation (PA)	0.876	0.914	0.679	0.502	0.641	0.468	0.454	0.824

Discriminant validity was assessed using the Fornell–Larcker criterion. As reported in Table 1, the square root of the AVE for each construct was greater than its corresponding correlations with other constructs, supporting adequate discriminant validity. Together, these results indicate that the measurement model demonstrates satisfactory reliability and validity, allowing for further evaluation of the structural model.

Discriminant validity was further assessed using the heterotrait–monotrait ratio of correlations (HTMT). As shown in Table 2, all HTMT values were below the conservative threshold of 0.85, indicating satisfactory discriminant validity among the study constructs (Henseler et al., 2015). These results provide additional support that the latent constructs are empirically distinct, thereby alleviating concerns related to construct overlap and multicollinearity in the structural model.

**TABLE 2:** Heterotrait-monotrait (HTMT) ratio.

Construct	AI	ER	CARQ	PC	PA
Athletic identity (AI)	–	–	–	–	–
Emotional resilience (ER)	0.536	–	–	–	–
Coach–athlete relationship quality (CARQ)	0.458	0.603	–	–	–
Peer climate (PC)	0.492	0.571	0.646	–	–
Psychological adaptation (PA)	0.588	0.742	0.561	0.548	v

Collinearity among predictor constructs was assessed using variance inflation factor (VIF) values to ensure that multicollinearity did not bias the estimated path coefficients. As reported in Table 3, all VIF values ranged from 1.286 to 1.472, which are well below the conservative threshold of 3.3 recommended for PLS-SEM analyses (Hair et al., 2021). These results indicate that collinearity is not a concern in the present study and that the structural model estimates can be interpreted with confidence.

**TABLE 3:** Variance inflation factor (VIF).

Predictor	Emotional resilience	Psychological adaptation
Athletic identity	1.286	1.354
Emotional resilience	–	1.472
Coach–athlete relationship quality	1.418	–
Peer climate	1.402	–

Table 4 presents the results of the structural model, examining the direct, indirect, and interaction effects proposed in the study. The findings indicate that athletic identity has a positive and statistically significant effect on emotional resilience (β = 0.421, *p* = 0.000), supporting H1. This result suggests that athletes who strongly define themselves through their athletic role are more likely to develop higher levels of emotional resilience, particularly in the context of injury-prone sports. Emotional resilience, in turn, exhibits a strong positive association with psychological adaptation (β = 0.534, *p* = 0.000), supporting H2 and underscoring its central role in facilitating adaptive psychological functioning under conditions of stress and injury risk.

**TABLE 4:** Structural model results.

Hypothesis	Path	β	t-value	*P*-value	95% CI (LL, UL)
H1	Athletic identity → emotional resilience	0.421	7.862	0.000s	[0.318, 0.521]
H2	Emotional resilience → psychological adaptation	0.534	9.418	0.000	[0.421, 0.639]
H3	Athletic identity → psychological adaptation	0.219	4.103	0.000	[0.113, 0.324]
H4	Athletic identity → emotional resilience → psychological adaptation (indirect effect)	0.225	6.487	0.000	[0.158, 0.298]
H5	Athletic identity × coach–athlete relationship quality → motional resilience	0.164	3.572	0.000	[0.071, 0.257]
H6	Athletic identity × peer climate → emotional resilience	0.142	3.118	0.002	[0.049, 0.231]

Athletic identity also demonstrates a direct positive effect on psychological adaptation (β = 0.219, *p* = 0.000), providing support for H3. The simultaneous significance of both the direct and indirect paths indicates a partial mediation pattern. This inference is further reinforced by the significant indirect effect of athletic identity on psychological adaptation via emotional resilience (β = 0.225, *p* = 0.000), supporting H4. The confidence interval for this indirect effect does not include zero, confirming the robustness of the mediating mechanism.

**FIGURE 2:**
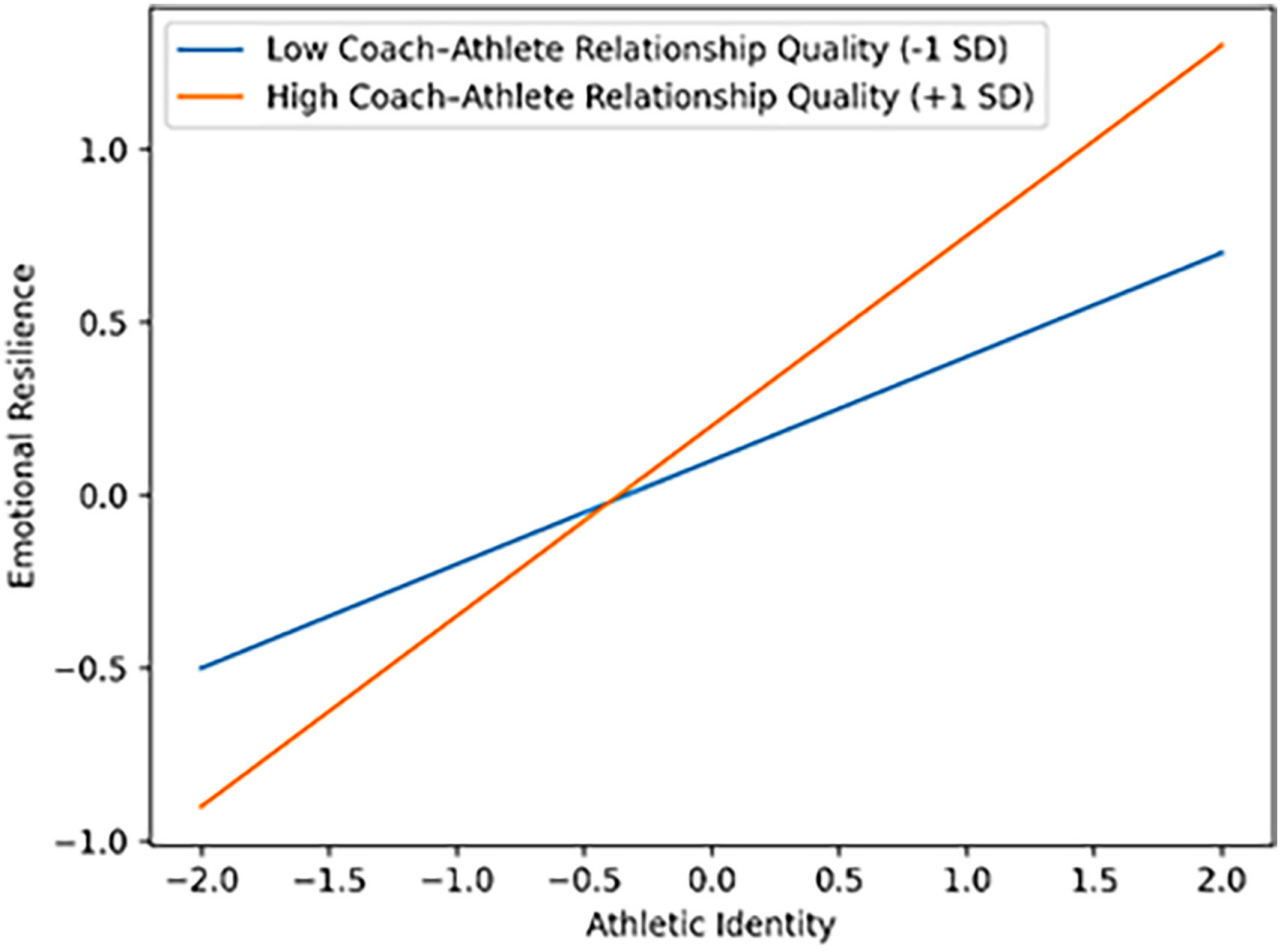
Simple slope analysis of coach-athlete relationship quality.

**FIGURE 3:**
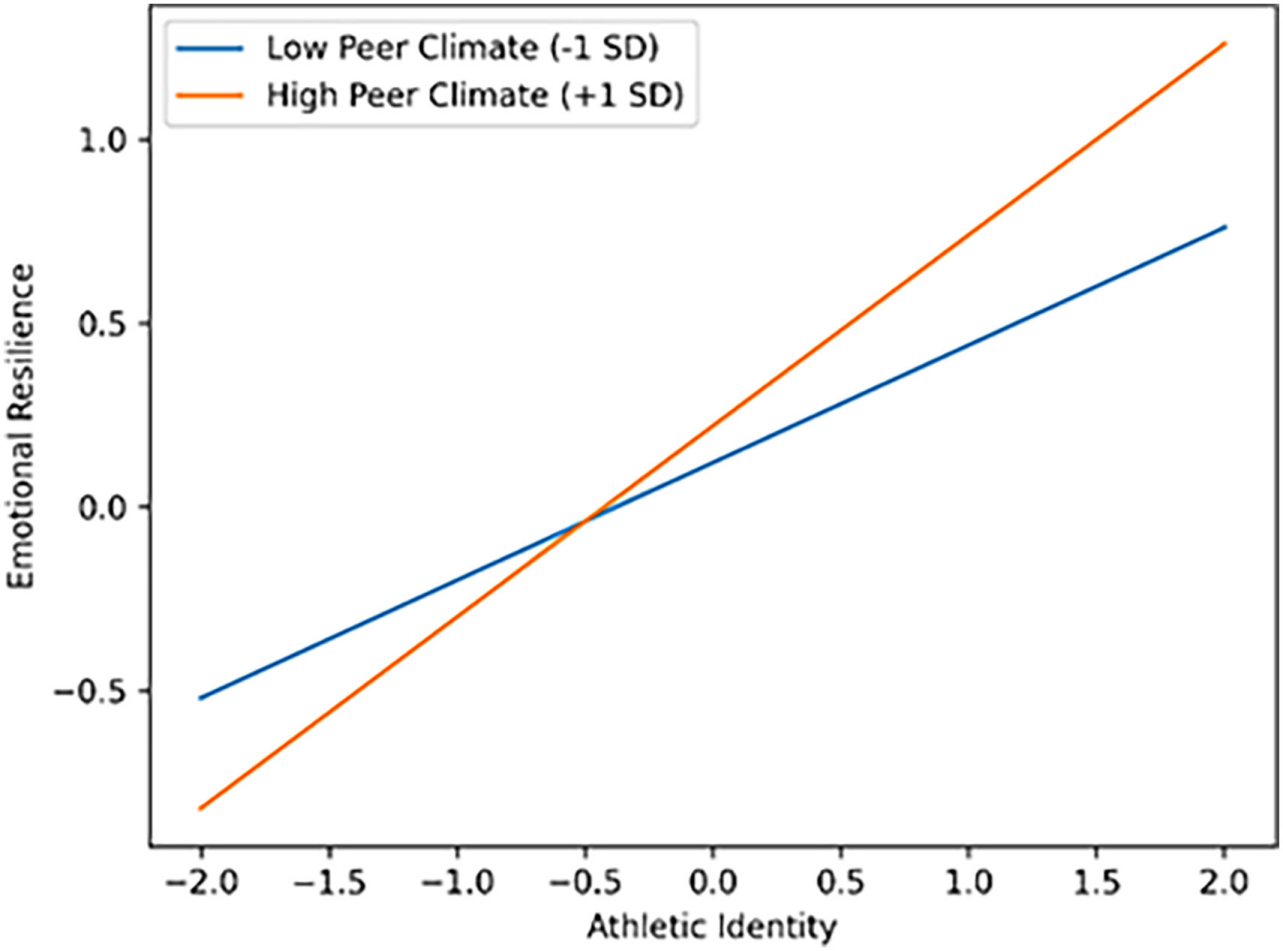
Simple slope analysis of peer climate.

In addition, the interaction effects reveal that social-contextual factors significantly shape the identity–resilience linkage. Specifically, coach–athlete relationship quality positively moderates the relationship between athletic identity and emotional resilience (β = 0.164, *p* = 0.000), supporting H5 (Figure 2). This finding indicates that the beneficial effect of athletic identity on emotional resilience is amplified when athletes perceive a high-quality relationship with their coach. Similarly, peer climate significantly moderates the same relationship (β = 0.142, *p* = 0.002), supporting H6, suggesting that supportive peer environments further strengthen the capacity of athletic identity to foster emotional resilience (Figure 3). Collectively, these results highlight that identity-based resilience processes are not uniform but depend substantially on the quality of athletes’ relational environments.

Table 5 reports the conditional indirect effects of athletic identity on psychological adaptation via emotional resilience at different levels of the moderators. The results show that the indirect effect remains positive and statistically significant across both low and high levels of coach–athlete relationship quality. However, the magnitude of the indirect effect is notably stronger at high levels of relationship quality (β = 0.279) compared to low levels (β = 0.171), indicating that emotionally supportive and collaborative coach–athlete relationships enhance the extent to which athletic identity translates into psychological adaptation through emotional resilience.

**TABLE 5:** Conditional indirect effects of athletic identity on psychological adaptation via emotional resilience.

Moderator	Level	Indirect effect (β)	t-value	*P*-value	95% CI (LL, UL)
Coach–athlete relationship quality	Low (−1 SD)	0.171	4.218	0.000	[0.093, 0.252]
	High (+1 SD)	0.279	6.002	0.000	[0.190, 0.376]
Peer climate	Low (−1 SD)	0.182	4.507	0.000	[0.106, 0.267]
	High (+1 SD)	0.261	5.736	0.000	[0.175, 0.355]

A similar pattern emerges for peer climate. The indirect effect of athletic identity on psychological adaptation via emotional resilience is significant at both low and high levels of peer climate, but the effect is stronger under more favorable peer conditions (β = 0.261) than under less supportive peer environments (β = 0.182). The non-overlapping confidence intervals across moderator levels provide further evidence that the strength of the mediating mechanism varies meaningfully as a function of the social context.

Taken together, the conditional indirect effects provide strong support for the proposed moderated mediation framework. These findings indicate that emotional resilience serves as a key psychological mechanism linking athletic identity to psychological adaptation, while coach–athlete relationship quality and peer climate act as critical boundary conditions that intensify this process. In injury-prone sport contexts, athletic identity is therefore most beneficial for psychological adaptation when embedded within supportive relational environments.

In addition, the explanatory power of the structural model was further assessed using R^2^ values for the endogenous constructs. The model explained a moderate proportion of variance in emotional resilience (R^2^ = 0.38) and a substantial proportion of variance in psychological adaptation (R^2^ = 0.61), indicating satisfactory predictive capability in accordance with recommended PLS-SEM guidelines (Hair et al., 2021). These findings suggest that the proposed framework demonstrates meaningful explanatory strength in predicting adaptive psychological functioning among athletes participating in injury-prone sports.

## 8 Discussion and implications

The present study aimed to examine how athletic identity contributes to psychological adaptation in injury-prone sports through emotional resilience and under varying relational conditions. Overall, the findings provide strong support for the proposed moderated mediation framework, indicating that athletic identity strengthens psychological adaptation both directly and indirectly through emotional resilience. Furthermore, the findings demonstrate that supportive coach–athlete relationships and positive peer climates strengthen the association between athletic identity and emotional resilience, thereby highlighting the critical role of social-contextual conditions in shaping adaptive psychological functioning among athletes participating in injury-prone sports.

In the present study, psychological adaptation is interpreted as athletes’ capacity to maintain positive emotional, psychological, and social functioning under conditions of injury risk, performance uncertainty, and ongoing sport-related stress. Consistent with prior adaptation research in sport psychology (Rubio et al., 2021; Samuel et al., 2024), the multidimensional wellbeing dimensions captured by the Mental Health Continuum–Short Form were considered reflective of adaptive psychological functioning within demanding sport environments.

Furthermore, the study advances understanding of psychological adaptation in injury-prone sports by explicating how athletic identity influences adaptive psychological functioning through emotional resilience and by identifying the social-contextual conditions under which this process is strengthened. Drawing on identity-based perspectives (Campo et al., 2019), conservation of resources theory (Hobfoll, 1989), and self-determination theory (Ryan and Deci, 2000), the findings provide empirical support for a multilevel process model in which individual self-concept, emotional regulation capacity, and relational environments jointly shape athletes’ psychological outcomes under conditions of heightened physical risk. The results offer consistent support for the hypothesized direct, mediating, moderating, and moderated mediation relationships, thereby extending existing sport psychology literature that has often examined these constructs in isolation.

Consistent with H1 and identity-based frameworks, the findings indicate that athletic identity positively contributes to athletes’ emotional resilience in injury-prone sport environments. This finding contributes to a long-standing debate in the sport psychology literature regarding whether athletic identity functions primarily as a psychological asset or liability (Brewer and Chatterton, 2024; Edison et al., 2021), particularly in injury contexts. A recent meta-analysis corroborates that earlier research has frequently emphasized the potential negative consequences of a strong athletic identity, such as identity foreclosure and emotional distress following injury (Lochbaum et al., 2022). However, more recent work suggests that athletic identity may also provide structure, purpose, and continuity, especially when athletes are supported by adaptive psychological and social resources (McGinley et al., 2022; Schmid et al., 2024). The present findings align with this emerging perspective by demonstrating that athletic identity can facilitate emotional resilience, suggesting that identification with the athlete role may foster emotional strength rather than vulnerability when athletes remain psychologically engaged with their sporting pursuits.

Supporting H2, the findings further underline the central role of emotional resilience in promoting psychological adaptation within high-risk sport environments. Emotional resilience has been conceptualized as an individual’s capacity to recover from stress and maintain psychological equilibrium in the face of adversity (Secer et al., 2025; Shinnick, 2024). In injury-prone sports, athletes are routinely exposed to uncertainty, pain, performance disruption, and fear of reinjury, all of which can undermine psychological functioning if not effectively managed. The present findings corroborate prior research linking resilience to psychological wellbeing and adaptive functioning in stressful contexts (Cheng and Jiao, 2024; Junnarkar et al., 2021; Powell et al., 2024), and extend this work by empirically situating emotional resilience as a key predictor of broader psychological adaptation among athletes operating under chronic injury risk.

Importantly, the mediating role of emotional resilience clarifies the psychological mechanism through which athletic identity contributes to psychological adaptation. Rather than exerting a purely direct influence, athletic identity appears to foster adaptive outcomes by enhancing athletes’ capacity to regulate emotions and recover from stress. This finding advances identity research by moving beyond direct-effect models and highlighting the importance of intermediary psychological processes. It also aligns with conservation of resources theory, which posits that individuals with access to valuable personal resources are better equipped to cope with stress and prevent resource loss (Hobfoll, 1989). Within this framework, athletic identity may function as a motivational resource that promotes emotional resilience, which in turn supports psychological adaptation when athletes encounter injury-related challenges.

Beyond mediation, the moderating effects of coach–athlete relationship quality and peer climate provide critical insight into the contextual boundaries of identity-based resilience processes. The significant interaction between athletic identity and coach–athlete relationship quality indicates that the positive effect of identity on emotional resilience is contingent upon the relational environment established by the coach. High-quality coach–athlete relationships, characterized by trust, commitment, and mutual respect, have been consistently linked to athletes’ motivation, wellbeing, and emotional functioning (Karayel et al., 2024; Zhang et al., 2023). From a self-determination theory perspective, such relationships are likely to satisfy athletes’ basic psychological needs for relatedness and competence, thereby enabling athletic identity to be expressed in adaptive rather than rigid or self-threatening ways (Ryan and Deci, 2000). The present findings extend this literature by demonstrating that coach–athlete relationship quality not only influences motivational outcomes but also shapes deeper emotional regulation capacities that underpin psychological adaptation.

Similarly, peer climate was found to moderate the relationship between athletic identity and emotional resilience, highlighting the importance of the broader team environment. Peer climates characterized by encouragement, shared learning goals, and emotional support appear to amplify the beneficial effects of athletic identity on resilience. This finding is consistent with research demonstrating that peer-created motivational climates influence athletes’ emotional experiences, wellbeing, and engagement (Li et al., 2025; Qiu et al., 2025). The present study extends this work by positioning peer climate as a critical boundary condition for identity-based emotional processes, suggesting that athletic identity is most psychologically adaptive when embedded within supportive peer systems rather than competitive or evaluative social environments.

The moderated mediation results represent one of the most novel contributions of the study. By demonstrating that the indirect effect of athletic identity on psychological adaptation via emotional resilience varies across levels of coach–athlete relationship quality and peer climate, the findings provide a nuanced, process-oriented account of psychological adaptation in injury-prone sports. To the authors’ knowledge, few studies have simultaneously examined identity, emotional resilience, and multiple relational moderators within a single empirical model. This integrative approach responds to calls for more complex and ecologically valid models of athlete psychology that account for interactions between individual and contextual factors (Lin et al., 2022; Schinke et al., 2024). The findings suggest that emotional resilience serves as a particularly powerful mechanism when athletes are embedded within relational environments that reinforce, rather than undermine, their athletic identity.

From a theoretical standpoint, the study contributes to several research streams. First, it extends athletic identity research by demonstrating that identity effects are conditional and mechanism-driven rather than uniformly adaptive or maladaptive. Second, it enriches resilience theory by situating emotional resilience within an identity–context framework, highlighting how resilience emerges from the interaction of personal and social resources. Third, it advances conservation of resources theory by empirically illustrating how relational resources enhance the utility of identity-based psychological resources under stress. Finally, the findings contribute to self-determination theory by underscoring the role of relational need satisfaction in enabling adaptive emotional regulation and psychological functioning.

The findings also carry important practical implications for athletes, coaches, and sport organizations operating in injury-prone contexts. Interventions aimed at promoting psychological adaptation should prioritize the development of emotional resilience rather than focusing exclusively on symptom reduction or injury-related distress. Psychological skills training programs that emphasize emotional recovery, adaptive coping, and emotional awareness may help athletes harness their athletic identity in psychologically beneficial ways. Coaches play a pivotal role in this process, and efforts to enhance coach–athlete relationship quality may yield substantial benefits for athletes’ emotional resilience and psychological adaptation. Coach education programs should therefore emphasize relational competencies alongside technical instruction, fostering environments that support athletes’ emotional needs during periods of injury risk.

The moderating role of peer climate further suggests that team-level interventions may be particularly effective. Creating team cultures that emphasize mutual support, shared growth, and learning from setbacks may strengthen the capacity of athletic identity to promote emotional resilience. Sport organizations may consider implementing peer mentorship programs or structured team-building initiatives designed to foster positive peer interactions. Such approaches align with contemporary athlete-centered models of sport development that emphasize holistic wellbeing alongside performance outcomes.

At the same time, not all athletes operate within highly supportive coaching or peer environments, particularly in individual sports or high-performance contexts characterized by limited interpersonal cohesion and elevated competitive pressure. Under such conditions, practitioners may need to place greater emphasis on individualized psychological support strategies, including resilience training, mental skills development, emotional self-regulation techniques, and access to sport psychology services. Even in the absence of strong relational support systems, interventions that strengthen athletes’ internal coping resources and adaptive self-regulation capacities may help sustain positive psychological functioning and reduce vulnerability to emotional distress in injury-prone sport settings.

Despite its contributions, the study has several limitations that should be acknowledged. The cross-sectional design precludes causal inference, and future research employing longitudinal or experimental designs would be better positioned to capture dynamic changes in identity, resilience, and psychological adaptation across injury and recovery trajectories. The reliance on self-report measures also raises concerns regarding common method variance, although the use of validated instruments mitigates this risk. Future studies could incorporate multi-source data, such as coach or peer reports, to strengthen measurement robustness. Although the study employed validated instruments and satisfactory validity indicators were observed, the use of self-reported data collected from the same respondents at a single point in time may still introduce potential common method bias. In addition, the peer climate instrument was originally developed within youth sport settings, and although the scale demonstrated acceptable psychometric performance in the present sample, future studies may further validate its applicability across broader adult and elite athletic populations. Additionally, while the study focused on injury-prone sports, the findings may not generalize to lower-risk or recreational sport contexts, warranting further investigation across diverse athletic populations.

Last but not the least, the cultural context of China may also have shaped the observed relationships in important ways. Chinese sport environments are often characterized by relatively collectivistic social norms, strong respect for authority figures, and heightened emphasis on interpersonal harmony and group cohesion, which may strengthen the psychological significance of coach–athlete relationships and peer climates in shaping athletes’ emotional functioning and adaptive responses. Consequently, the strength and nature of the observed relationships may differ across cultural settings characterized by more individualistic sport structures or different interpersonal dynamics. Future research should therefore examine the proposed framework across diverse cultural and athletic contexts to further evaluate the generalizability and cultural sensitivity of the present findings.

## 9 Conclusion

In conclusion, the present study offers a comprehensive, theory-driven account of how athletic identity contributes to psychological adaptation in injury-prone sports through emotional resilience and under specific social-contextual conditions. By integrating mediation and moderated mediation processes, the findings move beyond simplistic models and highlight the interdependence of identity, emotion, and relationships in shaping athletes’ psychological functioning. The results underscore that athletic identity is most psychologically beneficial when it fosters emotional resilience and is supported by high-quality coach–athlete relationships and positive peer climates. Together, these insights provide a robust foundation for future research and practice aimed at promoting adaptive psychological functioning in demanding sport environments.

## Data Availability

The original contributions presented in this study are included in this article/supplementary material, further inquiries can be directed to the corresponding author.
